# Evaluating the impact of genotype errors on rare variant tests of association

**DOI:** 10.3389/fgene.2014.00062

**Published:** 2014-04-01

**Authors:** Kaitlyn Cook, Alejandra Benitez, Casey Fu, Nathan Tintle

**Affiliations:** ^1^Department of Mathematics, Carleton CollegeNorthfield, MN, USA; ^2^Department of Applied Mathematics, Brown UniversityProvidence, RI, USA; ^3^Department of Mathematics, Massachusetts Institute of TechnologyBoston, MA, USA; ^4^Department of Mathematics, Statistics and Computer Science, Dordt CollegeSioux Center, IA, USA

**Keywords:** SKAT, gene-based, genotype uncertainty, misclassification, dosage

## Abstract

The new class of rare variant tests has usually been evaluated assuming perfect genotype information. In reality, rare variant genotypes may be incorrect, and so rare variant tests should be robust to imperfect data. Errors and uncertainty in SNP genotyping are already known to dramatically impact statistical power for single marker tests on common variants and, in some cases, inflate the type I error rate. Recent results show that uncertainty in genotype calls derived from sequencing reads are dependent on several factors, including read depth, calling algorithm, number of alleles present in the sample, and the frequency at which an allele segregates in the population. We have recently proposed a general framework for the evaluation and investigation of rare variant tests of association, classifying most rare variant tests into one of two broad categories (length or joint tests). We use this framework to relate factors affecting genotype uncertainty to the power and type I error rate of rare variant tests. We find that non-differential genotype errors (an error process that occurs independent of phenotype) decrease power, with larger decreases for extremely rare variants, and for the common homozygote to heterozygote error. Differential genotype errors (an error process that is associated with phenotype status), lead to inflated type I error rates which are more likely to occur at sites with more common homozygote to heterozygote errors than vice versa. Finally, our work suggests that certain rare variant tests and study designs may be more robust to the inclusion of genotype errors. Further work is needed to directly integrate genotype calling algorithm decisions, study costs and test statistic choices to provide comprehensive design and analysis advice which appropriately accounts for the impact of genotype errors.

## Introduction

Over the past 5 years, numerous gene-based tests of rare variant association have been proposed. Several summaries and reviews of these methods are available (Asimit and Zeggini, 2010; Bansal et al., 2010; Cooper and Shendure, 2011; Dering et al., 2011; Gibson, 2012). The majority of these tests accumulate evidence of genotype-phenotype association across multiple single nucleotide variants (SNVs) within a gene either by first collapsing genotypes of some or all of the SNVs (collapsing; burden; length tests) (Morgenthaler and Thilly, 2007; Li and Leal, 2008; Madsen and Browning, 2009; Han and Pan, 2010; Li et al., 2010; Morris and Zeggini, 2010; Zawistowski et al., 2010; Feng et al., 2011; Sul et al., 2011; Zhang et al., 2011; Dai et al., 2012) or by aggregating (e.g., summing) individual variant association statistics across all SNVs within a gene (variance components; joint tests) (Li and Leal, 2008; Basu and Pan, 2011; Ionita-Laza et al., 2011; Lin and Tang, 2011; Neale et al., 2011; Pan and Shen, 2011; Wu et al., 2011) (see Liu et al., 2013 for details).

A recent paper (Liu et al., 2013) introduced the terminology “length” and “joint” tests to illustrate a geometric interpretation of the gene-based, rare variant, test statistic formulation for case-control studies. Most rare variant test statistics can be written as functions of the generally stated Length (*L*_*p*_) or Joint (*J*_*p*_) test statistics as defined immediately below:

$$\begin{array}{l} \text{General Length Test Statistic, }L_{p}={\left(\sum_{i=1}^{m}{\left|\frac{c_{i}^{+}}{2N^{+}}\right|}^{p}\right)}^{1/p}\\ \;-{\left(\sum_{i=1}^{m}{\left|\frac{c_{i}^{-}}{2N^{-}}\right|}^{p}\right)}^{1/p}\\ \text{ General Joint Test Statistic, }J_{p}={\left(\sum_{i=1}^{m}{\left|\frac{c_{i}^{+}}{2N^{+}}-\frac{c_{i}^{-}}{2N^{-}}\right|}^{p}\right)}^{1/p} \end{array}$$

where, *m* is the number of SNVs within the gene, *N*^+^ and *N*^−^ indicate the sample sizes of the cases and controls, respectively, *c*^+^_*i*_ and *c*^−^_*i*_ indicate the observed number of minor alleles at variant *i*, within the case and control samples, respectively, and *p* reflects the choice of *L*^*p*^ norm. To date, most published length tests use *p* = 1, while most joint tests use *p* = 2. Thus, length tests compare the magnitudes (lengths) of the *m*-dimensional minor allele frequency (MAF) vectors between cases and controls by taking the *L*^*p*^ norms of the vectors, with larger differences in length indicating stronger evidence of genotype-phenotype association. Joint tests compare both the lengths of the case-control vectors, as well as the angle between the vectors (evidence for association increases as the magnitude of the angle between the vectors increases). This geometric framework provides the basis for theoretical evaluation of test behavior—moving beyond comparison of rare variant test statistic behavior solely by simulation.

Genotype errors occur when calling algorithms misidentify an individual's genotype (e.g., an individual who is actually AA is identified as AT). To date, the majority of evidence showing the detrimental effects of genotype error on this new class of rare variant tests has been based on simulation results. In particular, simulation of genotype data followed by simulation of genotype errors on those genotypes finds that the power of some specific length tests decreases—sometimes dramatically—in the presence of non-differential (independent of case-control status) genotyping errors. These power declines can be particularly large for errors misclassifying the common homozygote as the heterozygote, even when the error rate is relatively low (Powers et al., 2011). Relatedly, for some specific joint and length tests, the type I error rate increases above nominal levels in the presence of differential genotyping errors, even at low error rates. The magnitude of the type I error inflation increases further as the sample size, number of rare variants or relative difference in case-control error rates at the site increases, or as the MAF of variants decreases. Similarly, these effects are enhanced for errors from the common homozygote to the heterozygote (Mayer-Jochimsen et al., 2013). At error levels observed in sequence and imputed data for rare variants, the effects of errors on power and type I error can be measurable (Awadalla et al., 2010; Ilie et al., 2011; Nielsen et al., 2011; Rogers et al., 2014). These findings are similar to findings about the effects of both non-differential (Gordon et al., 2002, 2004; Kang et al., 2004a,b; Ahn et al., 2007) and differential (Moskvina et al., 2006; Ahn et al., 2009) errors when analyzed with single marker test statistics.

While such findings based on simulation are useful, their utility in providing a deeper understanding of the reasons why errors can be so detrimental to power and type I error is limited. In this paper, we use the geometric framework as a platform for deeper understanding of the mechanisms by which genotype errors impact rare variant tests of association. In particular, we use the geometric framework to gain greater insights into the relative impact of different types of genotype errors (homozygote to heterozygote, or vice versa), MAF, the differential or non-differential nature of the genotype errors and choice of rare variant test statistic on the power and type I error rate of length and joint tests.

## Methods

### Distributions, power and type I error rates of gene-based rare variant test statistics

We start by noting that *c*^+^_*i*_ ~ Binom(2*N*^+^, *f*^+^_*i*_) and *c*^−^_*i*_ ~ Binom(2*N*^−^, *f*^−^_*i*_), where *f*^+^_*i*_ and *f*^−^_*i*_ are the MAFs in the cases and controls, respectively. For a low prevalence disease, *f*^−^_*i*_ will be approximately equal to the population MAF, *f*_*i*_. We are often interested in the scaled difference of these counts, $D_{i}=\frac{c_{i}^{+}}{2N^{+}}-\frac{c_{i}^{-}}{2N^{-}}$. Applying basic distribution theory yields: *μ*_*D*_*i*__ = *f*^+^_*i*_ − *f*^−^_*i*_ and $\sigma_{D_{i}}^{2}=\frac{1}{2N^{+}}\left(f_{i}^{+}\left(1-f_{i}^{+}\right)\right)+\frac{1}{2N^{-}}\left(f_{i}^{-}\left(1-f_{i}^{-}\right)\right)$. For all rare variant tests considered in this manuscript, the null hypothesis is that *f*^+^_*i*_ = *f*^−^_*i*_ for all *i*. We start by stating assumptions needed for our analytic evaluation.

#### Assumptions

1. Let *ε*_01,*i*_ represent the probability that the major allele is misclassified as the minor allele at site *i*, and let *ε*_10,*i*_ represent the probability that the minor allele is misclassified as the major allele at site *i*. We can write the population MAF in both the cases and controls as a function of the true population minor allele frequencies and the error rate. In particular
  $$\begin{array}{l} f_{i}^{+*}=f_{i}^{+}\left(1-\varepsilon_{10,i}\right)+\left(1-f_{i}^{+}\right)\left(\varepsilon_{01,i}\right)\\ f_{i}^{-*}=f_{i}^{-}\left(1-\varepsilon_{10,i}\right)+\left(1-f_{i}^{-}\right)\left(\varepsilon_{01,i}\right) \end{array}$$
  where we assume that each allele has an equal chance of being misclassified and that likelihood of errors in the cases is the same as in the controls (non-differential errors). Differential errors follow a similar definition and assumption, except that the change of errors is different in cases and controls.
2. In all proofs and simulations, we assume that the allele frequencies in the population follow Hardy-Weinberg Equilibrium.
3. In all proofs and simulations, we assume that the variant sites within the gene are not in linkage disequilibrium (LD) as we have done in previous work (Mayer-Jochimsen et al., 2013). See the Discussion for implications.
4. When evaluating the impact of genotype errors on J^*^_2_ (Impact of Genotype Errors on the Type I Error and Power of J^*^_2_) and J^*^_∞_ (Impact of Genotype Errors on the Type I Error and Power of *J*_∞_), as well as when providing analytic power and sample size estimates (Asymptotic Power Formulas for L^*^_1_ and J^*^_2_), we explore the impact of genotype errors on the distributions of *c*^+^_*i*_ and *c*^−^_*i*_ as approximated by Normal distributions. In particular, that *c*^+^_*i*_ ⩪ Norm (*N*^+^*p*^+^_*i*_, *N*^+^*p*^+^_*i*_ (1 − *p*^+^_*i*_)) and *c*^−^_*i*_ ⩪ Norm (*N*^−^*p*^−^_*i*_, *N*^−^*p*^−^_*i*_ (1 − *p*^−^_*i*_)). It follows directly that *D*_*i*_ ⩪ Norm (*μ*_*D*_*i*__, σ^2^_*D*_*i*__), and, thus, $\frac{D_{i}^{2}}{\sigma_{D_{i}}^{2}}\dot{\sim }\chi_{1,\lambda }^{2}$ where $\lambda ={\left(\frac{\mu_{D_{i}}}{\sigma_{D_{i}}}\right)}^{2}$ is the non-centrality parameter. We evaluate robustness to this assumption as part of our simulation study (see Quality of Asymptotic Power and Type I Error Predictions).

#### Impact of genotype errors on the type I error and power of L^*^_1_

When *p* = 1, we can write

$$\begin{array}{l} L_{1}={\left(\sum_{i=1}^{m}{\left|\frac{c_{i}^{+}}{2N^{+}}\right|}^{p}\right)}^{\frac{1}{p}}-{\left(\sum_{i=1}^{m}{\left|\frac{c_{i}^{-}}{2N^{-}}\right|}^{p}\right)}^{\frac{1}{p}}\\ \;=\sum_{i=1}^{m}\frac{c_{i}^{+}}{2N^{+}}-\sum_{i=1}^{m}\frac{c_{i}^{-}}{2N^{-}}=\sum_{i=1}^{m}\left(\frac{c_{i}^{+}}{2N^{+}}-\frac{c_{i}^{-}}{2N^{-}}\right)=\sum_{i=1}^{m}D_{i} \end{array}$$

where we have dropped the absolute value since the observed minor allele counts will always be positive. Thus, $\mu \left(L_{1}\right)=\sum_{i,=\;1}^{m}\mu_{D_{i}}$ and $\sigma^{2}\left(L_{1}\right)=\sum_{i\;=\;1}^{m}\sigma_{D_{i}}^{2}$ when variant sites are independent (no LD).

When genotype errors are present (indicated by *), similar arguments hold. The distribution of $L_{1}^{*}=\sum_{i=1}^{m}\left(\frac{c_{i}^{+*}}{2N^{+}}-\frac{c_{i}^{-*}}{2N^{-}}\right)=\sum_{i=1}^{m}D_{i}^{*}$ has mean $\mu \left(L_{1}^{*}\right)=\sum_{i=1}^{m}\left(f_{i}^{+*}-f_{i}^{-*}\right)=\sum_{i=1}^{m}\mu_{D_{i}}^{*}$ and $\sigma^{2}\left(L_{1}^{*}\right)=\sum_{i=1}^{m}\sigma_{D_{i}^{*}}^{2}+2\sum_{i<j}Cov\left(D_{i}^{*},D_{j}^{*}\right)$ where, $\sum_{i=1}^{m}\sigma_{D_{i}^{*}}^{2}=\sum_{i=1}^{m}\left(\frac{1}{2N^{+}}\right)(f_{i}^{+*}(\text{​​​​​​​​​}1-f_{i}^{+*}))+\frac{1}{2N^{-}}\left(f_{i}^{-*}\left(1-f_{i}^{-*}\right)\right)$. As above, when variant sites are independent (no LD) ∑_*i < j*_ *Cov* (D^*^_i_, D^*^_j_) = 0.

***Non-differential genotype errors and the type I error rate***. When the null hypothesis is true, it is straightforward to see that *μ* (*L*_1_) = 0. When there are non-differential genotype errors $\mu \left(L_{1}^{*}\right)=\sum_{i\;=\;1}^{m}\mu_{D_{i}}^{*}=0$ since *f*^+*^_i_ − *f*^−*^_i_ = *f*^+^_*i*_ (1 − *ε*_10,*i*_) + (1 − *f*^+^_*i*_) (*ε*_01,*i*_) − *f*^−^_*i*_ (1 − *ε*_10,*i*_) − (1 − *f*^−^_*i*_) (*ε*_01,*i*_) = (1 − *ε*_10,*i*_) (*f*^+^_*i*_ − *f*^−^_*i*_) − (*ε*_01,*i*_) (*f*^+^_*i*_ − *f*^−^_*i*_) = 0 for all *i*. Bross (1954) proved that estimates of the variance of D^*^_i_ are unbiased in the presence of non-differential misclassification errors for both small and large samples. Thus, linear scaled sums of these estimates (as in L^*^_1_) are also unbiased, resulting in a test which controls the Type I error rate.

***Non-differential genotype errors and power***. Given the fact that the Type I error is maintained in the presence of non-differential errors, we now explore the impact of non-differential genotype errors on the power of L^*^_1_. To do this we start by noting that *μ* (L^*^_1_) can be written as:

$$\begin{array}{l} \mu \text{​}\left(L_{1}^{*}\right)=\sum_{i=1}^{m}\left(\left(1-\varepsilon_{10,i}\right)\left(f_{i}^{+}-f_{i}^{-}\right)-\left(\varepsilon_{01,i}\right)\left(f_{i}^{+}-f_{i}^{-}\right)\right)\\ \;=\sum_{i=1}^{m}\left(\left(1-\varepsilon_{10,i}-\varepsilon_{01,i}\right)\left(f_{i}^{+}-f_{i}^{-}\right)\right) \end{array}$$

Thus, in the presence of non-differential genotype errors (*ε*_10,*i*_ > 0, *ε*_01,*i*_ > 0), *μ* (D^*^_i_) = (1 − *ε*_10,*i*_ − *ε*_01,*i*_) (*f*^+^_*i*_ − *f*^−^_*i*_) < *μ* (*D*_*i*_) = (*f*^+^_*i*_ − *f*^−^_*i*_), moving *μ* (D^*^_i_) closer to 0 (which is our expectation under the null hypothesis), with both *ε*_10,*i*_ and *ε*_01,*i*_ contributing equally to the shift of the mean of the alternative distribution closer to 0. When *f*^+^_*i*_ ≥ *f*^−^_*i*_ for all i (all variants are non-causal or risk increasing), then $\left(L_{1}^{*}\right)<\mu \left(L_{1}\right)=\sum_{i=1}^{m}\left(f_{i}^{+}-f_{i}^{-}\right)$, moving *μ* (L^*^_1_) closer to 0 (which is our expectation under the null hypothesis). When at least one *f*^+^_*i*_ < *f*^−^_*i*_ (at least one protective variant), then moving *μ* (D^*^_i_) closer to 0, will increase the overall value of *μ* (L^*^_1_) since there will be less “cancellation” occurring between risk increasing and risk reducing variants when computing the test statistic.

We will now show that in general, σ^2^(L^*^_1_) > σ^2^(*L*_1_). Recall that $\sigma^{2}\left(L_{1}\right)=\sum_{i=1}^{m}\sigma_{D_{i}}^{2}$ and that $\sigma_{D_{i}}^{2}=\frac{1}{2N^{+}}\left(f_{i}^{+}\left(1-f_{i}^{+}\right)\right)+\frac{1}{2N^{-}}\left(f_{i}^{-}\left(1-f_{i}^{-}\right)\right)$, with similar relationships true when errors are present (denoted by *). To show that σ^2^(L^*^_1_) > σ^2^(*L*_1_) it is sufficient to show that σ^2^_D^*^_i__ > σ^2^_*D*_*i*__ for all *i*, an inequality which is true when both *f*^+*^_i_ (1 − *f*^+*^_i_) > *f*^+^_*i*_ (1 − *f*^+^_*i*_) and *f*^−*^_i_ (1 − *f*^−*^_i_) > *f*^−^_*i*_ (1 − *f*^−^_*i*_).

To see that *f*^+*^_i_ (1 − *f*^+*^_i_) > *f*^+^_*i*_ (1 − *f*^+^_*i*_) is true in most cases consider that 0 < *f*^+^_*i*_ < 0.5 and, thus, in most situations, 0 < *f*^+*^_i_ < 0.5 because we have defined *f* as the MAF. Thus, *f*^+*^_i_ (1 − *f*^+*^_i_) > *f*^+^_*i*_ (1 − *f*^+^_*i*_) when *f*^+*^_i_ > *f*^+^_*i*_, an inequality that will be true in most practical cases, as shown below

$$\begin{array}{l} f_{i}^{+*}>f_{i}^{+}f_{i}^{+}-f_{i}^{+}\left(\varepsilon_{10,i}+\varepsilon_{01,i}\right)+\varepsilon_{01,i}>f_{i}^{+}\\ \;\varepsilon_{01,i}>f_{i}^{+}\left(\varepsilon_{10,i}+\varepsilon_{01,i}\right)\\ \;\varepsilon_{01,i}>\varepsilon_{10,i}\left(\frac{f_{i}^{+}}{1-f_{i}^{+}}\right)\approx \varepsilon_{10,i}f_{i}^{+} \end{array}$$

Where we make use of the fact that for rare alleles *f* is quite small, and so, unless the value of *ε*_10,*i*_ is many orders of magnitude larger than *ε*_01,*i*_ the inequality will be true. Similar arguments hold when showing *f*^−*^_i_ (1 − *f*^−*^) > *f*^−^_*i*_ (1 − *f*^−^_*i*_).

It is also important to note that the increases to σ^2^(L^*^_1_) due to effect of *ε*_01_ are substantially more than the effects of *ε*_10_. This can be seen by observing that *f*^+*^_i_ = *f*^+^_*i*_ − *f*^+^_*i*_ (*ε*_10,*i*_ + *ε*_01,*i*_) + *ε*_01_ = *f*^+^_*i*_ (1 − *ε*_10,*i*_) + (1 − *f*^+^_*i*_) *ε*_01,*i*_. Since *f*_*i*_ is small, increases in values of *ε*_01,*i*_ increase variance, while increases to *ε*_10,*i*_ decrease variance, but substantially less. Increases in variance, combined with shifting of the mean of the alternative distribution toward the mean of the null distribution, will result in decreases in power. The only exception is in cases where genotype errors occur on protective variants, which, as shown in the previous section, may mitigate power loss to some extent. Our evaluation shows that the relative effects of *ε*_01,*i*_ on power loss are more than power loss driven by *ε*_10,*i*_.

***Differential genotype errors and the type I error rate***. Differential genotype errors occur when the genotype error rate in the cases (*ε*^+^) is different than it is in the controls (*ε*^−^). In this case, it follow directly from earlier arguments that,

$$\begin{array}{l} \mu \text{​}\left(L_{1}^{*}\right)=\sum_{i=1}^{m}f_{i}^{+}\left(1-\varepsilon_{10,i}^{+}\right)+\left(1-f_{i}^{+}\right)\varepsilon_{01,i}^{+}-(f_{i}^{-}\left(1-\varepsilon_{10,i}^{-}\right)\\ \;+\left(1-f_{i}^{-}\right)\varepsilon_{01,i}^{-}) \end{array}$$

Where, + and − indicate the different genotype error rates in the cases and controls, respectively. We note that when the null hypothesis is true, the following is true for each variant *i*.

$$\begin{array}{l} f_{i}\text{​}\left(1-\varepsilon_{10,i}^{+}\right)+\left(1-f_{i}\right)\varepsilon_{01,i}^{+}-\left(f_{i}\text{​}\left(1-\varepsilon_{10,i}^{-}\right)+\left(1-f_{i}\right)\varepsilon_{01,i}^{-}\right)\\ \;=f_{i}\text{​}\left(\varepsilon_{10,i}^{-}-\varepsilon_{10}^{+},i\right)+\left(1-f_{i}\right)\left(\left(\varepsilon_{01,i}^{+}-\varepsilon_{01,i}^{-}\right)\right) \end{array}$$

This quantity is not zero in the presence of differential genotype errors. This means that when differential genotype errors are present *μ* (L^*^_1_) ≠ 0, which is sufficient to show that the resulting type I error rate will typically no longer be the nominal value. The exception is when the effects of differential genotype errors cancel out, which can occur if genotype error rates are larger in the cases for some variants, and larger in the controls for other variants. Examining the equation further suggests that in general the larger the difference in error rates, the larger the type I error rate will be, with differences in the *ε*_01,*i*_ error rates contributing more to inflation in the type I error rate than differences in the *ε*_10,*i*_ error rates, since differences in *ε*_10,*i*_ only impact *μ* (L^*^_1_) through a term which is multiplied by *f*, typically a small quantity. Sites with higher MAF (larger *f*_*i*_) will tend to increase the value of *μ* (L^*^_1_) more, however, the impact is scaled by the difference in case and control genotyping error rates, which will typically be a small quantity, meaning that the overall impact of *f*_*i*_ on the value of *μ* (L^*^_1_) is quite minimal.

Much of the argument about the relationship between σ^2^(L^*^_1_) and σ^2^(*L*_1_) in the presence of differential genotype errors follows directly from arguments made in the previous section (Power) when examining non-differential errors. To show that, in general, σ^2^(L^*^_1_) > σ^2^(*L*_1_) it is sufficient to show that σ^2*^_D_*i*__ > σ^2^_*D*_*i*__, an inequality which is true when both *f*^+*^_i_ (1 − *f*^+*^_i_) > *f*^+^_*i*_ (1 − *f*^+^_*i*_) and *f*^−*^_i_ (1 − *f*^−*^_i_) > *f*^−^_*i*_ (1 − *f*^−^_*i*_). It is typically true that *f*^+*^_i_ (1 − *f*^+*^_i_) > *f*^+^_*i*_ (1 − *f*^+^_*i*_) because $\varepsilon_{01,i}^{+}>\varepsilon_{10,i}^{+}\left(\frac{f_{i}^{+}}{1-f_{i}^{+}}\right)\approx \varepsilon_{10}^{+}f_{i}^{+}$, with similar arguments holding in the controls—even when the error rates in the controls are different than in the cases. Thus, once again, the effect of *ε*_01_ on the variance is substantially more than the effect of *ε*_10_. Since increases in variance will result in increases in the type I error rate, *ε*_01_ has a potentially large impact on the type I error rate, while *ε*_10_ has less impact (really only impacting *μ* (L^*^_1_).

#### Impact of genotype errors on the type I error and power of J^*^_2_

When *p* = 2, we can write

$$\begin{array}{l} J_{2}={\left(\sum_{i=1}^{m}{\left|\frac{c_{i}^{+}}{2N^{+}}-\frac{c_{i}^{-}}{2N^{-}}\right|}^{2}\right)}^{1/2}=\sqrt{\sum_{i=1}^{m}{\left(\frac{c_{i}^{+}}{2N^{+}}-\frac{c_{i}^{-}}{2N^{-}}\right)}^{2}}\\ \;=\sqrt{\sum_{i=1}^{m}{\left(D_{i}\right)}^{2}} \end{array}$$

Thus, $\mu \left(J_{2}^{2}\right)=\sum_{i=1}^{m}\mu_{D_{i}^{2}}=\sum_{i=1}^{m}{\left(f_{i}^{+}-f_{i}^{-}\right)}^{2}$ and $\sigma^{2}\left(J_{2}^{2}\right)=\sum_{i=1}^{m}\sigma_{D_{i}^{2}}^{2}+2\sum_{i<j}Cov\left(D_{i}^{2},D_{j}^{2}\right)$, where *Cov* (*D*^2^_*i*_, *D*^2^_*j*_) is the covariance between the differences in case and control allele counts at variant i and j, and, thus, is an indirect measure of LD. When variant sites are independent (no LD) *Cov* (*D*^2^_*i*_, *D*^2^_*j*_) = 0.

When genotype errors are present (indicated by *), similar arguments hold. The distribution of $J_{2^{*}}^{2}=\sum_{i=1}^{m}{\left(\frac{c_{i}^{+*}}{2N^{+}}-\frac{c_{i}^{-*}}{2N^{-}}\right)}^{2}=\sum_{i=1}^{m}{\left(D_{i}^{*}\right)}^{2}$ has mean $\mu \left(J_{2^{*}}^{2}\right)=\sum_{i=1}^{m}\mu_{D_{i}^{2}}^{*}$ and $\sigma^{2}\left(J_{2^{*}}^{2}\right)=\sum_{i=1}^{m}\sigma_{D_{i}^{*}{}^{2}}^{2}+2\sum_{i<j}Cov\left(D_{i}^{*}{}^{2},D_{j}^{{*}^{2}}\right)$. As above, when variant sites are independent (no LD) ∑_*i < j*_ *Cov* (D^*^2^^_*i*_, D^*^2^^_*j*_) = 0.

Insights into the direction and pattern of effects of genotype errors on *J*^2^_2_ are aided by utilizing χ^2^ distributions. As noted in Distributions, Power and Type I Error Rates of Gene-based Rare Variant Test Statistics (Assumptions), $\frac{D_{i}^{2}}{\sigma_{D_{i}}^{2}}\dot{\sim }\chi_{1,\lambda }^{2}$ where $\lambda ={\left(\frac{\mu_{D_{i}}}{\sigma_{D_{i}}}\right)}^{2}$ is the non-centrality parameter. It follows directly that $J_{2,scaled}^{2}=\sum_{i=1}^{m}{\left(\frac{D_{i}}{\sigma_{D_{i}}}\right)}^{2}\dot{\sim }\chi_{m,\lambda }^{2}$ where $\lambda =\sum_{i=1}^{m}{\left(\frac{\mu_{D_{i}}}{\sigma_{D_{i}}}\right)}^{2}$ is the non-centrality parameter. Our analyses focus on the behavior of *J*^2^_2,*scaled*_ which can be interpreted as a MAF-variant weighted version of *J*_2_ in the spirit of Madsen and Browning (2009) and others.

***Non-differential genotype errors and the type I error rate***. When the null hypothesis is true, $\lambda =\sum_{i=1}^{m}{\left(\frac{\mu_{D_{i}}}{\sigma_{D_{i}}}\right)}^{2}=\sum_{i=1}^{m}{\left(\frac{f_{i}^{+}-f_{i}^{-}}{\sigma_{D_{i}}}\right)}^{2}=0$. This is also true in the presence of non-differential genotype errors since, as shown in Non-differential Genotype Errors and the Type I Error Rate, *f*^+*^_i_ − *f*^−*^_i_ = 0 for all *i*, and so $\lambda^{*}=\sum_{i=1}^{m}{\left(\frac{f_{i}^{+*}-f_{i}^{-*}}{\sigma_{D_{i}}^{*}}\right)}^{2}=0$. Thus, the type I error rate is maintained since the distribution of *J*^2^_2,*scaled*_ is the same with or with non-differential genotype errors when the null hypothesis is true.

***Non-differential genotype errors and power***. When the alternative hypothesis is true, *f*^+^_*i*_ ≠ *f*^−^_*i*_ for at least one *i*, and the non-centrality parameter, $\lambda =\sum_{i=1}^{m}{\left(\frac{f_{i}^{+}-f_{i}^{-}}{\sigma_{D_{i}}}\right)}^{2}$ will be greater than 0. Furthermore, the power of J^*^_2,*scaled*_ (non-differential genotype errors) will be lower than *J*_2,*scaled*_ (no errors) if λ^*^ < λ. As shown in 2.1.1.2, σ^*^_D_*i*__ > σ_*D*_*i*__, and so we can show that, in general, λ^*^ < λ if (*f*^+*^_i_ − *f*^−*^_i_)^2^ < (*f*^+^_*i*_ − *f*^−^_*i*_)^2^ is also true, which is the case since (*f*^+*^_i_ − *f*^−*^_i_)^2^ = (1 − *ε*_10,*i*_ − *ε*_01,*i*_)^2^ (*f*^+^_*i*_ − *f*^−^_*i*_)^2^ < (*f*^+^_*i*_ − *f*^−^_*i*_)^2^. Furthermore, we can conclude that the impact of the errors follows the same pattern as for L^*^_1_, namely that the relative effects of *ε*_01,*i*_ on power loss are more than power loss driven by *ε*_10,*i*_.

***Differential genotype errors and the type I error rate***. When differential genotype errors are present, then there may be inflation of the type I error rate. This inflation occurs because, due to differential genotype errors, the non-centrality parameter, $\lambda^{*}=\sum_{i=1}^{m}{\left(\frac{f_{i}^{+*}-f_{i}^{-*}}{\sigma_{D_{i}}^{*}}\right)}^{2}$, is no longer, necessarily, zero. This result follows directly from the fact that *f*^+*^_i_ may not equal *f*^−*^_i_ for all *i*, since *f*^+*^_i_ − *f*^−*^_i_ = *f*_*i*_ ((*ε*^−^_10,*i*_ − *ε*^+^_10_, *i*) + (*ε*^−^_01,*i*_ − *ε*^+^_01,*i*_)) + (*ε*^+^_01,*i*_ − *ε*^−^_01,*i*_) will not necessarily equal 0. even when *f*^+^_*i*_ = *f*^−^_*i*_ = *f*. Following directly from Differential Genotype Errors and the Type I Error Rate, the case-control differences in the *ε*_01,*i*_ error rates will inflate the type I error rate more than case-control differences in the *ε*_10,*i*_.

#### Impact of genotype errors on the type I error and power of *J*_∞_

Liu et al. (2013) showed that, while under-explored in the literature, the choice of norm for both Length and Joint statistics had practical implications. In particular, as the value of the norm increases, gene-based rare variant tests are increasingly robust to the inclusion of non-causal variants (i.e., variants for which *f*^+^_*i*_ = *f*^−^_*i*_). To explore how the impact of genotype errors may vary based on choice of norm, we consider using the infinity norm on a joint test. Following Liu et al. (2013), we let, $J_{\infty }=\begin{array}{c} \begin{array}{l} argmax\\ 1\leq i\leq m \end{array} \end{array}\left(\frac{c_{i}^{+}}{2N^{+}}-\frac{c_{i}^{-}}{2N^{-}}\right).$

***Non-differential genotype errors and the type I error rate***. Results earlier showed that the Type I error rate is maintained because when non-differential genotype errors are present *μ*_*D*_*i*__ = μ^*^_D_*i*__ = 0, and that estimates of the variance of D^*^_i_ are also unbiased resulting in a test (J^*^_∞_) which maintains the type I error rate since the distribution at each variant site maintains the type I error rate and the variant sites are independent of each other.

***Non-differential genotype errors and power***. When there are non-differential genotyping errors, the power will be reduced because μ^*^_D_*i*__ < *μ*_*D*_*i*__. However, because *J*_∞_ focuses only on a single variant site (namely, the site, *i*, showing the largest difference in minor allele frequencies), the impact of errors on power relative to *L*_1_ and *J*_2_ may be lessened because the power loss does not accumulate across variant sites when genotype errors are evenly distributed across variant sites. However, if non-differential genotype errors are focused only on the sites with the largest true difference in minor allele counts power loss may be substantial. The relative impact of *ε*_01_ and *ε*_10_ follow patterns described earlier (Non-differential Genotype Errors and Power).

***Differential genotype errors and the type I error rate***. When differential genotyping errors are present, the type I error rate will increase because μ^*^_D_*i*__ ≠ 0. As with power, the impact on type I error may be lessened because the type I error effects do not accumulate across variant sites when genotype errors are evenly distributed across variant sites. However, if the differential genotype errors are contained only on a single variant—inducing the largest observed differences in minor allele frequencies—the type I error rate may inflate above levels observed for *L*_1_ and *J*_2_. The relative impact of *ε*_01_, *ε*_10_ and *f* follow patterns described in Differential Genotype Errors and the Type I Error Rate.

### Asymptotic power formulas for L^*^_1_ and J^*^_2_

We can derive general power and sample size formulas for situations of both differential and non-differential errors, which yields the potential for directly computing the change in power and sample size increase necessary to mitigate the effects of genotype errors.

#### L^*^_1_

As established in the introduction to Section Distributions, Power and Type I Error Rates of Gene-based Rare Variant Test Statistics, the minor allele counts *c*^+^_*i*_ and *c*^−^_*i*_ are both binomially distributed, with *c*^+^_*i*_ ~ Binom(2*N*^+^, *f*^+^_*i*_) and *c*^−^_*i*_ ~ Binom(2*N*^−^, *f*^−^_*i*_). While not needed in our initial exploration of the direction and relative effects of non-differential genotype errors on the type I error rate and power, to make predictions of the actual change in power or type I error rate, we utilize the normal approximation described earlier (Distributions, Power and Type I Error Rates of Gene-based Rare Variant Test Statistics Assumptions).

Since $D_{i}\dot{\sim }\text{Norm}\left(\mu_{D_{i}},\sigma_{D_{i}}^{2}\right),L_{1}=\sum_{i=1}^{m}D_{i}\dot{\sim }\text{Norm}(\sum_{i=1}^{m}\mu_{D_{i}},\sum_{i=1}^{m}\sigma_{D_{i}}^{2})$. In the presence of errors, $L_{1}^{*}=\sum_{i=1}^{m}D_{i}^{*}\dot{\sim }\text{Norm}\left(\sum_{i=1}^{m}\mu_{D_{i}^{*}},\sum_{i=1}^{m}\sigma_{D_{i}^{*}}^{2}\right)$.

***Estimated power in the presence of non-differential genotype error***. To determine the test's power, first find the *z*_1 − *α*_ quantile, *C*, under the null hypothesis as $C=z_{1-\alpha }\sqrt{\sum_{i=1}^{m}\sigma_{D_{i,H_{0}}}^{2}}$. Find the corresponding quantile, *z*_*β*_, on the alternative hypothesis distribution as $z_{\beta }=\frac{C-\sum_{i=1}^{m}\mu_{D_{i,H_{A}}^{*}}}{\sqrt{\sum_{i=1}^{m}\sigma_{D_{i,H_{A}}^{*}}^{2}}}$ and compute the power, π, as π = 1 − Φ (*z*_*β*_) where Φ (·) is the normal cdf.

***Sample size necessary in the presence of non-differential genotype error***. Since power decreases in the presence of non-differential genotype error (as shown in Non-differential Genotype Errors and Power), we can find the sample size necessary for a given power in the presence of genotype errors. To assist in the following proof, let *k* = *N*^−^/*N*^+^ = *N*^−*^/*N*^+*^ and $t_{i}^{*}=\left(\frac{1}{2}\right)\left(f_{i}^{+*}\left(1-f_{i}^{+*}\right)+\frac{1}{k}f_{i}^{-*}\left(1-f_{i}^{-*}\right)\right)$ so that $\sigma_{D_{i}^{*}}^{2}=\frac{1}{2N^{+*}}\left(f_{i}^{+*}\left(1-f_{i}^{+*}\right)\right)+\frac{1}{2kN^{+*}}\left(f_{i}^{-*}\left(1-f_{i}^{-*}\right)\right)=\frac{t_{i}^{*}}{N^{+*}}$.

To determine *N*^+*^ needed for a given *α* and *β* note that

$$\begin{array}{l} z_{\beta }=\frac{C-\sum_{i=1}^{m}\mu_{D_{i,H_{A}}^{*}}}{\sqrt{\sum_{i=1}^{m}\frac{t_{i,H_{A}}^{*}}{N^{+*}}}}=\frac{z_{1-\alpha }\sqrt{\sum_{i=1}^{m}\frac{t_{i,H_{0}}^{*}}{N^{+*}}}-\sum_{i=1}^{m}\mu_{D_{i,H_{A}}^{*}}}{\sqrt{\sum_{i=1}^{m}\frac{t_{i,H_{A}}^{*}}{N^{+*}}}}\\ \;z_{1-\alpha }\sqrt{\sum_{i=1}^{m}t_{i,H_{0}}^{*}}-z_{\beta }\sqrt{\sum_{i=1}^{m}t_{i,H_{A}}^{*}}=\frac{\sum_{i=1}^{m}\mu_{D_{i,H_{A}}^{*}}}{\sqrt{\frac{1}{N^{+*}}}} \end{array}$$

And so,

$$N^{+*}={\left(\frac{z_{1-\alpha }\sqrt{\sum_{i=1}^{m}t_{i,H_{0}}^{*}}-z_{\beta }\sqrt{\sum_{i=1}^{m}t_{i,H_{A}}^{*}}}{\sum_{i=1}^{m}\mu_{D_{i,H_{A}}^{*}}}\right)}^{2}$$

To find the percent sample size increase necessary to maintain power, simply compute the ratio of *N*^+*^ to *N*^+^, where *N*^+^ is determined following the same procedure as is used for *N*^+*^, only using values for *t*_*i*_ and *μ*_*D*_*i*__ not in the presence of errors.

***Type I error rate in the presence of differential genotype error***. In the presence of differential error, we can use a similar procedure to the one described in Estimated Power in the Presence of Non-Differential Genotype Error to determine the Type I error rate. Specifically, first find the *z*_1 − *α*_ quantile, *C*, under the null hypothesis as $C=z_{1-\alpha }\sqrt{\sum_{i=1}^{m}\sigma_{D_{i,H_{0}}}^{2}}$ corresponding to the nominal type I error rate *α*. Find the corresponding type I error rate in the presence of differential genotype errors, *z*_1 − α_^*^, as $z_{1-\alpha^{*}}=\frac{C-\sum_{i=1}^{m}\mu_{D_{i,H_{0}}^{*}}}{\sqrt{\sum_{i=1}^{m}\sigma_{D_{i,H_{0}}^{*}}^{2}}}$ and compute the inflated type I error rate, 1 − Φ (*z*_1 − α_^*^) where Φ (·) is the normal cdf.

#### J^*^_2_

In Section Impact of Genotype Errors on the Type I Error and Power of j^*^_2_ we demonstrated that $J_{2,scaled}^{2}=\sum_{i=1}^{m}{\left(\frac{D_{i}}{\sigma_{D_{i}}}\right)}^{2}\dot{\sim }\chi_{m,\lambda }^{2}$ where $\lambda =\sum_{i=1}^{m}{\left(\frac{\mu_{D_{i}}}{\sigma_{D_{i}}}\right)}^{2}$ is the non-centrality parameter. The non-centrality parameter can be used to find the power, type I error rate and related quantities.

***Estimated power in the presence of non-differential genotype error***. To determine the test's power, first find *C* = χ^2^_*m*,*α*_. Then, find the value of *β* such that *C* = χ^2^_*m*, *β*, λ^*^_ and compute the power, π, as π = 1 − *β* and where λ^*^ is the non-centrality parameter in the presence of non-differential genotype errors.

***Sample size necessary in the presence of non-differential genotype error***. Since power decreases in the presence of non-differential genotype error (as shown in Non-differential Genotype Errors and Power), we can find the sample size necessary to attain a particular level of power in the presence of genotype errors. As was done in Sample Size Necessary in the Presence of Non-Differential Genotype Error, we will focus on obtaining the percent increase in sample size necessary (*N*^+*^/*N*^+^) when genotype errors are present to maintain power when genotype errors are not present, where we again let *k* = *N*^−^/*N*^+^ = *N*^−*^/*N*^+*^ and $t_{i}^{*}=\left(\frac{1}{2}\right)\left(f_{i}^{+*}\left(1-f_{i}^{+*}\right)+\frac{1}{k}f_{i}^{-*}\left(1-f_{i}^{-*}\right)\right)$ so that $\lambda =\sum_{i=1}^{m}{\left(\frac{\mu_{D_{i}}}{\sigma_{D_{i}}}\right)}^{2}=\sum_{i=1}^{m}\left(\frac{\mu_{D_{i}}{}^{2}}{\frac{t_{i}}{N^{+}}}\right).$.

We start by noting that in order to maintain power, the value of the non-centrality parameter without errors, λ^*^, must be the same as the value of the non-centrality parameter when errors are present, λ^*^.

Thus, we solve the following for *N*^+*^/*N*^+^.

$$\begin{array}{l} \;\lambda =\lambda^{*}\\ \sum_{i=1}^{m}\left(\frac{\mu_{D_{i}}{}^{2}}{t_{i}}\right)=\sum_{i=1}^{m}\left(\frac{\mu_{D_{i}^{*}}{}^{2}}{\frac{t_{i}^{*}}{N^{+*}}}\right)\\ \;\frac{N^{+*}}{N^{+}}=\frac{\sum_{i=1}^{m}\left(\frac{\mu_{D_{i}}{}^{2}}{t_{i}}\right)}{\sum_{i=1}^{m}\left(\frac{\mu_{D_{i}^{*}}{}^{2}}{t_{i}^{*}}\right)} \end{array}$$

***Type I error rate in the presence of differential genotype error***. In the presence of differential error, we can use a similar procedure to the one described in Estimated Power in the Presence of Non-Differential Genotype Error to determine the Type I error rate. To determine the test's power, first find *C* = χ^2^_*m*,*α*_, the nominal type I error rate with no errors. Then, find the value of α^*^ (the inflated type I error) such that *C* = χ^2^_*m*,α^*^_, λ^*^ where λ^*^ is the non-centrality parameter in the presence of differential genotype errors.

### Simulation

We conducted a simulation study In order to determine to confirm theoretical intuitions described above, evaluate the quality of asymptotic normal distributions and to demonstrate that, while not explicitly considered above, joint and length test behavior across a wider class of norms (*L*_1_, *L*_2_, *L*_4_, *L*_∞_, *J*_1_, *J*_2_, *J*_4_, *J*_∞_) follows predicted patterns.

#### Simulation settings

For all simulation settings we consider a situation where there were 1000 cases and 1000 controls, and the number of variants, *m*, was fixed at 8. Genotypes at each variant, *i*, were simulated independently, following the assumptions of Hardy–Weinberg Equilibrium in the controls. Genotype errors were added to the true genotypes according to three error different models: *ε*_10_ error only, *ε*_01_ error only, and both *ε*_10_ and *ε*_01_ errors. Due to the stringent priors often placed on genotype callers, calling rare minor alleles is difficult, and thus *ε*_01_ error rates tend to be smaller than *ε*_10_ error rates (Powers et al., 2011). In order to reflect these realistic differences in error rates, we considered the following seven error settings, which are given as (*ε*_01_, *ε*_10_): (0, 0), (0, 0.1), (0, 0.5), (0.01, 0), (0.05, 0), (0.01, 0.1), (0.05, 0.5). We considered five different MAF settings: all variants MAF = 1%, all variants MAF = 0.1%, all variants MAF = 0.01%, two variants at 1%/six variants at 0.1% and two variants at 1%/six variants at 0.01%. All 35 combinations of MAF and genotype error rates were then considered for additional situations using differential and non-differential errors.

For non-differential errors, we used a relative risk distribution of 1.5 for MAF = 1%, 3 for MAF = 0.1% and 5 for MAF = 0.01% for risk-increasing, and the inverse for protective variants with those MAFs. We then considered six different mixes of causal and non-causal variants (1) all variants non-causal, (2) all variants risk increasing, (3) all variants risk reducing, (4) ½ variants risk reducing and ½ risk increasing, (5) ½ variants non-causal and ½ risk increasing, and (6) ½ variants non-causal, ¼ risk increasing and ¼ risk reducing), for a total of 6 × 35 = 210 settings with non-differential errors, 35 of which have no risk variants. In the case of differential errors, the relative risk was set to 1, and two different magnitudes of differential error were considered: relative difference in case and control genotype error rates (error rate in cases divided by error rate in controls) of 1.2, 1.5, 1/1.2, and 1/1.5. Thus, we considered 35 × 4 = 140 different cases of differential genotyping error.

A follow-up simulation study was conducted for the purposes of better understanding the behavior of tests with different norms. In particular, we started with the same 35 combinations of MAF and genotype error rate as in the main simulation study. We then considered two settings: one with 8 SNPs and the other with 16 SNPs, where in each case only one SNP in the set was causal (designated to be a SNP with a larger MAF in cases where SNPs have varying MAF). This simulation only considered non-differential error.

#### Calculating power and type I error

For each simulation setting listed above, we generated 1000 independent samples. We then used phenotype permutation (1000 permutations for each sample) to compute *p*-values for eight different test statistics: *L*_1_, *L*_2_, *L*_4_, *L*_∞_, *J*_1_, *J*_2_, *J*_4_, *J*_∞_, where the *p*-value is the percent of permuted values of the test statistic that exceeded the observed value. The power or type I error rate is then computed as the percentage of the 1000 samples with *p*-values less than 0.05. For *L*_1_ and *J*_2_ asymptotic power predictions were also computed for each setting.

## Results

### Overall impacts of non-differential errors

#### Type I error is control in the presence of non-differential errors

There were 35 simulation settings where there were no causal variants and non-differential genotype errors. To assess the overall control of the type I error rate, we looked at all 280 simulation by test statistic combinations (35 settings × 8 different statistics). An empirical type I error rate between 3 and 7% was considered to be reasonable control of the type I error rate (nominal level = 5%; approximate 99% margin of error = 2%). The vast majority (86.1%; 241/280) of test-statistic combinations showed reasonable control of the nominal type I error rate (empirical type I error rate between 3 and 7%). Of the 39 remaining settings, all showed deflation of the empirical type I error rate below the nominal level (Mean = 0.01, *SD* = 0.011, Min. = 0, Max. = 0.028). Twenty-five of the thirty-nine settings occurred when all variants had MAF = 0.01%, meaning that the average number of rare variants in the gene being analyzed was only 1.6 in the cases and 1.6 in the controls across all 8 variant sites combined. Across the remaining 14 settings, the average MAF was still relatively low (mean = 0.0011). The 39 settings were fairly indiscriminate across the 8 different test statistics considered here. Overall, type I error was controlled in the presence of non-differential errors.

#### Non-differential genotype errors decrease power

To assess the overall relationship between non-differential genotype errors and power when causal variants were present, we regressed empirical power on (a) average MAF across all variants, (b) magnitude of errors (0,1,5; where for *ε*_10_, 0 = 0%, 1 = 1%, and 5 = 5% and for *ε*_01_, 0 = 0%, 1 = 10%, and 5 = 50%), (c) percent of risk increasing variants and (d) percent of risk reducing variants for each of the test statistic by type of error (*ε*_01_ only, *ε*_10_ only, or *ε*_01_ and *ε*_10_) combinations where at least one variant increased or reduced disease risk. Overall, when focusing on the impact of genotype errors, we found that regression model coefficients for *ε*_01_ only and *ε*_01_ and *ε*_10_ models were quite similar, while *ε*_10_ only was quite different. This confirms that the impact of *ε*_10_ is much less than that of *ε*_01_. Furthermore, as error rates increased, power decreased (e.g., 3–5% for 1% increase in *ε*_01_ errors). Finally, as expected, increases to the MAF and percent of risk increasing variants increased power (e.g., increase in average MAF of 0.1%, increased power 1.0–3.2%; increase of 10% in proportion of risk increasing variants increased power 1.3–7.0%), while increases to the percent of risk-reducing variants increased power for joint tests (0.5–2.4%) and decreased power (0.6–1.3%) for length tests. Table 1 shows the coefficients for regression models across all non-differential genotype error settings.

**Table 1:** **Regression model coefficients relating power loss/gain to simulation parameters**.

**Norm**	**Type**	**Error magnitude (per 1% for *ε*_**01**_; 10% for *ε*_10_)^a^**			**MAF (per 0.1%)^b^**			**Percent risk increasing (per 10%)^c^**			**Percent risk reducing (per 10%)^d^**		
		***ε*_01_ only**	***ε*_10_ only**	***ε*_01_ and *ε*_10_**	***ε*_01_ only**	***ε*_10_ only**	***ε*_01_ and *ε*_10_**	***ε*_01_ only**	***ε*_10_ only**	***ε*_01_ and *ε*_10_**	***ε*_01_ only**	***ε*_10_ only**	***ε*_01_ and *ε*_10_**
1	Length	−0.04^**^	−0.02^**^	−0.05^***^	0.015^**^	0.010	0.011^*^	0.053^***^	0.070^***^	0.046^**^	−0.007	−0.010	−0.006
	Joint	−0.05^***^	−0.04^***^	−0.05^***^	0.028^***^	0.031^***^	0.023^***^	0.034^***^	0.049^***^	0.030^***^	0.017^*^	0.024^**^	0.015^*^
2	Length	−0.04^**^	−0.03^**^	−0.05^***^	0.017^**^	0.014^**^	0.013^*^	0.050^***^	0.062^***^	0.043^***^	−0.009	−0.011	−0.007
	Joint	−0.05^***^	−0.04^***^	−0.05^***^	0.028^***^	0.032^***^	0.024^***^	0.030^***^	0.040^***^	0.026^**^	0.014^*^	0.018^*^	0.012
4	Length	−0.04^**^	−0.03^***^	−0.05^***^	0.021^***^	0.020^***^	0.017^***^	0.045^***^	0.054^***^	0.039^***^	−0.009	−0.013	−0.008
	Joint	−0.04^***^	−0.03^***^	−0.05^***^	0.024^***^	0.027^***^	0.019^***^	0.023^***^	0.029^***^	0.020^**^	0.009	0.012	0.008
8	Length	−0.03^***^	−0.03^***^	−0.04^***^	0.017^**^	0.018^***^	0.014^***^	0.028^***^	0.039^***^	0.026^***^	−0.010	0.011	−0.007
	Joint	−0.03^***^	−0.03^***^	−0.03^***^	0.017^***^	0.020^***^	0.014^***^	0.015^***^	0.017^***^	0.013^**^	0.005	0.005	0.005

* p < 0.05;

** p < 0.01;

*** *p < 0.001*.

a *Increase in power for a 1% increase in error rate for *ε*_01_ or a 10% increase in error rate for *ε*_10_. For example, for J_2_, in the presence of *ε*_01_ only errors, a 1% increase in genotype error rate decreases power by an average of 5% points*.

b *Increase in power for a 0.1% increase in average MAF across all variant sites in the gene. For example, for J_2_, in the presence of *ε*_01_ only errors, a 0.1% increase in average MAF across all variant sites increases power by an average of 2.8% points*.

c *Increase in power for a 10% point increase in the number of risk increasing SNPs. For example, for J_2_, in the presence of *ε*_01_ only errors, a 10% increase in number of number of risk increasing SNPs across all variant sites increases power by an average of 3.4% points*.

d *Increase in power for a 10% point increase in the number of risk reducing SNPs. For example, for J_2_, in the presence of *ε*_01_ only errors, a 10% increase in number of number of risk reducing SNPs across all variant sites increases power by an average of 1.7% points*.

**Figure 1:**
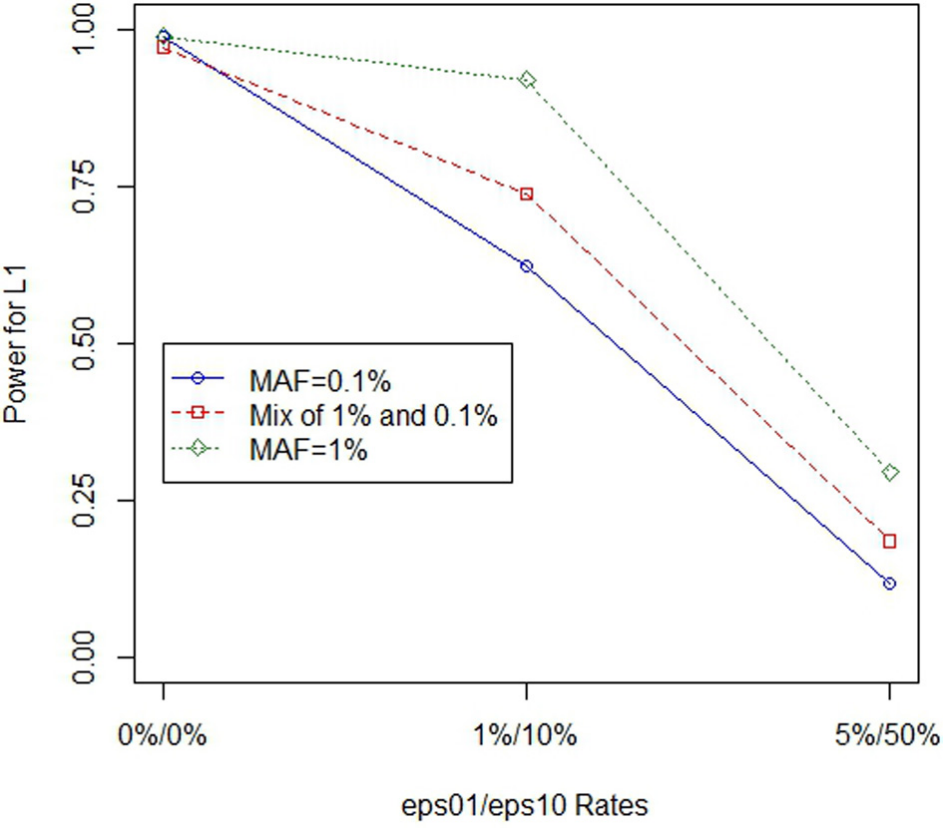
**Power for *L*_1_ in the presence of non-differential genotype errors at different MAF**. Power loss occurs when non-differential genotype errors are present at a locus. The power curves illustrated are at a site with eight causal variants. As genotype errors increase, power loss occurs. However, the power loss is most substantial when the minor allele frequency is the lowest.

Figure 1 further illustrates that the effect of genotype errors is compounded by the MAF. While the power is similar when no errors are present, similar magnitude errors for lower MAF decrease power at a faster pace than in cases with larger MAF variants.

### Overall impacts of differential errors

**Figure 2:**
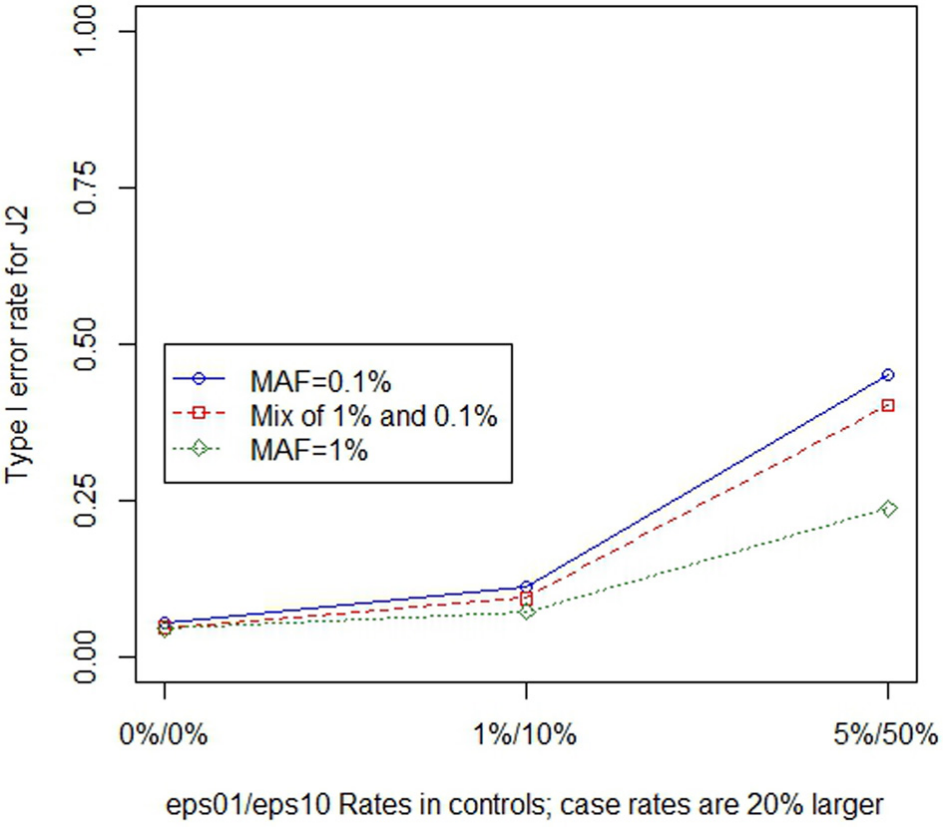
**Type 1 error rate for *J*_2_ in the presence of differential genotype errors at different MAF**. As differential error rates increase, the type I error rate increases. The type I error rate is illustrated at a site with eight non-causal variants. As differential (20% higher in cases) genotype error rates increased, the type I error rate increased. When the MAF was low, this effect was even larger.

Similar to the previous section, we used regression to assess the overall impacts of the three main simulation parameters (MAF, error magnitude and ratio of case to control errors) on the type I error rate when there were differential genotype errors. Table 2 shows the coefficients for regression models across all differential genotype error settings. In general, regression coefficients are similar for the *ε*_01_ only and *ε*_01_ and *ε*_10_ models, confirming that, as is the case for non-differential genotype errors, the effect of *ε*_10_ errors are less compared to the effects of *ε*_01_ errors. When *ε*_01_ errors are present, the type I error rate increased when increasing either the magnitude of the errors (between 6 and 13% increase in type I error rate for 1% increase in *ε*_01_ errors) or increasing the difference between the case and control error rates (between 9 and 12% increase in type I error rate for 10% relative increase in case error rate); changes to the MAF alone did not had little impact the type I error rate. However, as MAF decreases the effects of differential genotyping errors become even greater in magnitude, as illustrated in Figure 2 for *J*_2_, but a pattern that is true regardless of choice of test statistic.

**Table 2:** **Regression model coefficients relating type I error loss/gain to simulation parameters**.

**Norm**	**Type**	**Error magnitude (per 1% for *ε*_01_; 10% for *ε*_10_)^a^**			**MAF (per 0.1%)^b^**			**Ratio of case and control error rates (per 10%)^c^**		
		***ε*_01_ only**	***ε*_10_ only**	***ε*_01_ and *ε*_10_**	***ε*_01_ only**	***ε*_10_ only**	***ε*_01_ and *ε*_10_**	***ε*_01_ only**	***ε*_10_ only**	***ε*_01_ and *ε*_10_**
1	Length	0.08^***^	0.01^*^	0.07^***^	−0.004	0.008^*^	−0.005	0.12^***^	−0.004	0.11^***^
	Joint	0.13^***^	0.02^**^	0.12^***^	−0.005	0.009^**^	−0.009	0.10^***^	0.022^*^	0.10^***^
2	Length	0.08^***^	0.01^*^	0.07^***^	−0.003	0.007^*^	−0.005	0.11^***^	−0.006	0.11^***^
	Joint	0.13^***^	0.02^**^	0.12^***^	−0.005	0.009^**^	−0.009	0.10^***^	0.022^*^	0.10^***^
4	Length	0.08^***^	0.01^*^	0.08^***^	−0.002	0.007^*^	−0.004	0.10^***^	−0.005	0.10^***^
	Joint	0.12^***^	0.01^**^	0.11^***^	−0.004	0.008^**^	−0.008	0.10^***^	0.020^*^	0.09^***^
8	Length	0.06^***^	0.005	0.06^***^	−0.001	0.004^*^	−0.003	0.10^***^	−0.003	0.10^***^
	Joint	0.10^***^	0.01^**^	0.10^***^	−0.003	0.005^**^	−0.007	0.09^***^	0.013^*^	0.09^***^

* p < 0.05;

** p < 0.01;

*** *p < 0.001*.

a *Increase in type I error rate for a 1% increase in error rate for *ε*_01_ or a 10% increase in error rate for *ε*_10_. For example, for J_2_, in the presence of *ε*_01_ only errors, a 1% increase in genotype error rate increases the average type I error rate by 13% points when differential genotype errors are present*.

b *Increase in type I error rate for a 0.1% increase in average MAF across all variant sites in the gene. For example, for J_2_, in the presence of *ε*_10_ only errors, a 0.1% increase in average MAF across all variant sites increases the average type I error rate by 0.9% points*.

c *Increase in type I error for a 10% increase in the relative difference between the ratio of the case to control error rate. For example, for J_2_, in the presence of *ε*_01_ only errors, if the ratio of case error rate is 10% larger than control error rate 10% (e.g., 0.011 and 0.01), then the average type I error rate increases by 10% points*.

### The impact of genotype errors on choice of test statistic

While we have described the general effects of genotype errors on power and type I errors within particular test statistics, the geometric framework provides a basis for comparisons about the effects of genotype errors across two characteristics of rare variant test statistic: choice of length or joint test and choice of norm. We now consider each of these choices in turn.

#### Choice of length or joint test statistic

As shown both theoretically and validated by simulation, the general patterns of the effects of genotype error and allele frequency on length and joint tests are similar (see Methods, Overall Impacts of Non-Differential Errors, and Overall Impacts of Differential Errors). However, there is one important distinction worth addressing. In particular, recall the distinction between length and joint tests: length tests use the difference in case-control total allele frequency at the locus as the statistic, while joint tests compute the difference in allele frequencies at each variant site and then sum the differences across the locus.

***Non-differential errors***. For non-differential errors at a causal locus, if genotype errors yield a reduction in the difference in the cumulative MAF between cases and controls, there will be power loss. For joint tests, if genotype errors yield a reduction in the cumulative differences in allele frequency, there will be power loss. Thus, for joint tests, total power loss is a straightforward cumulative function of the power loss at each variant site. Things are, however, more complex for length tests. In a situation where all variants are risk-increasing, total power loss is a cumulative function of the power loss at each variant site. However, length tests lose power when protective variants and risk-increasing variants are present in the same gene because the effects of the variants “cancel out.” In this case, genotype errors can mitigate some of the power loss due to cancellation by bringing the difference in case-control allele counts closer together at protective variant sites (see Section non-Differential Genotype Errors and Power for details).

***Differential errors***. Similar to Non-Differential Errors, the effects of differential genotype errors on joint tests is simply the accumulation of the effects at each variant site. However, the effect of differential errors on length tests becomes more complex. For example, if *ε*_10_ is larger in the cases than in the controls for a risk increasing variant, then differential errors can create a variant site which has more rare alleles in the controls than in the cases increasing the type I error rate for both length and joint tests. However, for length tests, the inflation of the type I error rate may be mitigated if a protective variant is present in the gene or if another variant in the gene has *ε*_10_ is larger in the controls than in the cases. Details follow directly from equations in Section Differential Genotype Errors and the Type I Error Rate.

#### Choice of norm

While the focus of the bulk of literature has been on development of *L*_1_ or *J*_2_ tests, recent work has shown potential advantages to the use of higher normed tests as a built in form of variant weighting which may yield higher power, while controlling the type I error rate when the proportion of non-causal variants is high. We will now explore the simulation results by evaluating the performance of test statistics using different norms.

**Figure 3:**
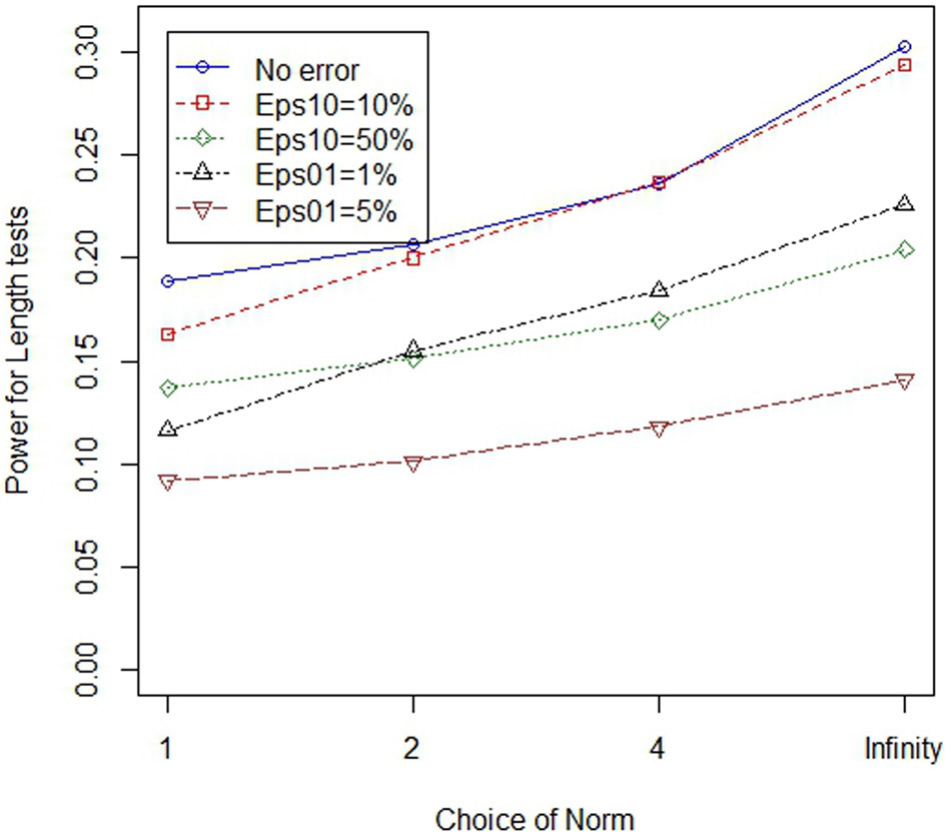
**Higher norms are more robust to genotype errors when the proportion of non-causal variants is larger: length tests**. The figure illustrates power of four different (norm) length statistics, under varying error models. All test statistics experience power loss in the presence of errors. However, power loss can be mitigated through the use of high norm test statistics.

**Figure 4:**
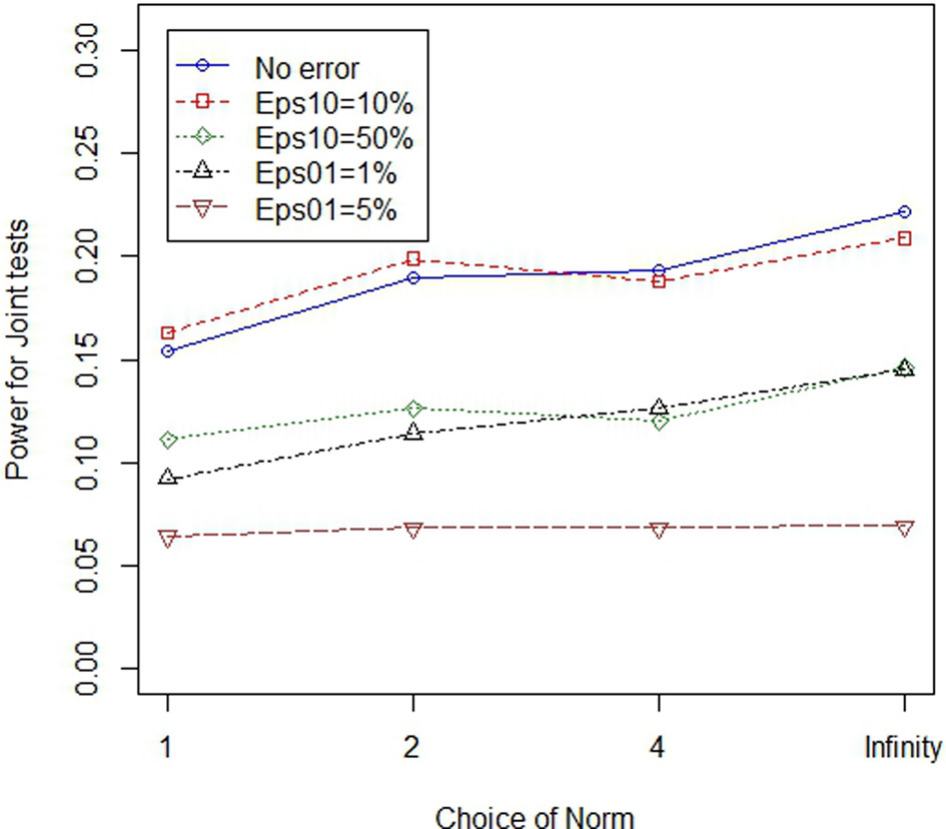
**Higher norms are more robust to genotype errors when the proportion of non-causal variants is larger: joint tests**. The figure illustrates power of four different (norm) joint statistics, under varying error models. All test statistics experience power loss in the presence of errors. However, power loss can be mitigated through the use of high norm test statistics.

In the main simulation, lower normed tests always outperformed higher normed tests in the main simulation where there were 8 variants, with either 50 or 100% of the variants classified as “causal” in cases where at least one variant at the locus modified disease risk. In the follow-up simulation we considered situations with 8 and 16 variants, where only one of the variants modified risk. When only one of the eight variants was causal, low norm tests outperformed high norm tests. However, when only one of sixteen variants was causal, high normed tests outperformed low norm tests in some cases. Figures 3, 4 illustrate the general patterns for length and joint tests, across norms. In short, while genotype errors contributed to power loss, the power loss was partially mitigated through the use of the larger norm.

### Quality of asymptotic power and type I error predictions

In order to evaluate the quality of asymptotic power and type I error predictions we compared the predicted power and type I error rates (see Simulation) to those obtained via permutation in the simulation study for ***L***_**1**_ and ***J***_**2**_. We use a significance level of 5% to evaluate consistency of predictions, but a follow-up analysis using lower significance thresholds of 10^−4^, 10^−5^, and 10^−6^ for a select group of simulation settings showed similar levels of consistency with predicted power and type I error rates as described in the following three sections (detailed results shown).

#### Type I error predictions in the presence of non-differential genotype error

As expected the type I error rate of the three asymptotic tests generally matched permutation tests since the asymptotic tests predicted 5% type I error rate in all cases (details not shown) and the permutation tests generally demonstrated control of the type I error rate, except in cases of extremely low (aggregate) MAF (see Type I Error is Control in the Presence of Non-Differential Errors for details), where the permutation tests showed empirical type I error rates less than the nominal level.

#### Power predictions in the presence of non-differential genotype error

Overall, predicted power was very close to observed power. Across 175 simulation settings with causal variants, most power predictions were within 10% of the true power (91% for ***L***_**1**_, and 83% for ***J***_**2**_). The quality of power predictions was strongly associated with the average MAF across the 8 sites in the control sample, as shown in Table 3.

**Table 3:** **Proportion of simulation settings and average MAF, within each absolute difference subcategory**.

**Abs. Diff**.		**Differential error**		**Non-differential error**	
		***L*_1_**	***J*_2_**	***L*_1_**	***J*_2_**
<0.05	Percentage of settings (Count/Total)	98.9% (137/140)	83.6% (117/140)	88.0% (154/175)	73.1% (128/175)
	Mean control MAF	0.6%	0.6%	0.6%	0.7%
0.05–0.1	Percentage of settings (Count/Total)	2.1% (3/140)	13.6% (19/140)	5.7% (10/175)	15.4% (27/175)
	Mean control MAF	0.01%	0.1%	0.3%	0.2%
>0.1	Percentage of settings (Count/Total)	0	2.9% (4/140)	6.3% (11/175)	11.4% (20/175)
	Mean control MAF	–	0.3%	0.004%	0.1%

#### Type I error predictions in the presence of differential genotype error

Similarly, predicted type I error inflation from differential genotype errors was very close to the empirical type I error rate across 140 simulation settings with no risk variants, but differential genotype errors present. The vast majority of type I error predictions were within 5% of the empirical type I error rate (91% for ***L***_**1**_ and 84% for ***J***_**2**_). Again, the quality of predictions was strongly associated with the average MAF in the control sample.

#### Software

Software (R scripts) for asymptotic power predictions and sample size computations for ***L***_**1**_ and ***J***_**2**_ based on the formulas and methods shown in Asymptotic Power Formulas for L^*^_1_ and J^*^_2_ is provided on the research group's website at: http://www.dordt.edu/statgen and following the links to the Software page.

## Discussion

Misclassification errors are a perennial problem in data analysis, and can be particularly magnified when using new technology which is often more error prone than mature technology. Recently, there has been substantial methodological effort devoted to the development of methods for analyzing next-generation sequencing data. However, much of this effort has ignored the problem of misclassification errors in the underlying genotype data (genotype errors). We have demonstrated that the persistent issue of genotype errors in next-generation sequencing data (Nielsen et al., 2011; Browning and Browning, 2013) has the potential to substantially reduce power and/or increase the type I error rate of the majority of related rare variant tests of association. Researchers should use the software and analytic tools described above to easily estimate the impact of genotype errors on downstream analyses. Thus, appropriately increasing sample size of next-generation studies to minimize power loss due to genotype error.

We have provided an initial theoretical justification behind recent simulation results evaluating the impact of both non-differential and differential genotype errors. In particular, we have confirmed that errors from the common homozygote to the heterozygote (*ε*_01_) are particularly detrimental. The effects are further compounded depending upon whether the genotype errors are differential (increasing MAF increases type I error rate) or non-differential (decreasing MAF decreases power). In general, the effects of heterozygote to common homozygote errors (*ε*_10_) are small and varied. The type I error rate is maintained in the presence of non-differential misclassification errors, with some over-conservatism when using permutation tests with extremely small allele frequencies due to the discrete nature of the permutation distribution. However, the type I error rate inflates in the presence of differential genotype errors. Our results are shown explicitly for common classes of test statistics, but are suggestive of the impact of genotype errors on all tests within the broad classes of length and joint tests regardless of the norm chosen.

To better understand why common homozygote to heterozygote errors can be so detrimental, it is useful to consider how many misclassifications are actually occurring in a dataset of interest. In the case of non-differential genotype errors, when examining rare variants (*p* is small), even small values of *ε*_01_ can yield many errors because most individuals in the dataset are common homozygotes. For example, on average, in a sample of 10,000 individuals, a rare variant with population MAF, *p* = 0.001, 9990 individuals will be the common homozygote, and so if *ε*_01_ is only 0.01, we expect nearly 100 (0.01^*^9990) misclassifications. On the other hand, even if *ε*_10_ is large (e.g., *ε*_10_ = 0.10), this yields, on average a small number of misclassifications (e.g., 0.10 * 10 = 1). Notably, due to the aggregating nature of all gene-based rare variant tests as compared to single marker tests, the effects of genotype errors aggregate across variant sites within the gene, further increasing impact on power loss and type I error inflation.

Liu et al. (2013) demonstrated that the use of larger norms in rare variant tests provides increased robustness to the inclusion of non-causal variants. Our analysis demonstrates that another advantage of these tests is that they may be more robust to genotype errors than lower normed tests. Rare variant tests using a larger norm place increasing weight on sites with larger MAF in the cases or controls (length tests) or on the difference in MAF between cases and controls (joint tests). Because of the cumulative nature of the impact of genotype errors on rare variant tests, use of higher norms, reduces the overall impact of genotype errors. Whether high norm (e.g., infinity norm) tests are a powerful choice in practice is dependent upon underlying genetic architecture, dependent upon what percent of the variants at the locus are, in fact, causal, and how much prior understanding of the potential functional implications of those variants (e.g., synonymous vs. non-synonymous) can be used to minimize the impact of non-causal variants on the test (e.g., only including non-synonymous variants in the test). Importantly, in cases where genotype errors are larger for some variants, if the largest observed effects are at sites with a low error rate, and non-causal SNPs have a higher error rate, high normed tests may perform particularly well. Of course, high-normed tests perform less optimally compared to low-normed tests when numerous causal variants are present. Thus, use of methods in the spirit of those proposed by Derkach et al. (2013) have the potential to combine high norm tests with low-normed tests to yield a combined testing approach which is robust and powerful to numerous genetic architectures and genotype error distributions. Continued exploration of this class of high-normed rare variant tests is needed to assess its practical utility.

A related issue is that nearly all rare variant tests proposed to date do not explicitly account for genotype errors in the formulation of the test statistic. However, inclusion of genotype errors in the test statistic may also help to mitigate power loss and type I error inflation from genotype errors. While use of higher norms may, in some cases, mitigate the impact of genotype errors, development of tests which explicitly incorporate errors into the test may perform even better. There are some recently developed methods which address these weaknesses by directly incorporating sequence quality information (Daye et al., 2012) or advocating pooled study designs (Wang et al., 2012; Navon et al., 2013). However, in general, these methods remain outside of the mainstream. Expanded consideration of the impact of errors on more commonly used methods, combined with increased use of methods which explicitly model errors and/or study designs which limit the impact of errors are needed.

To explicitly incorporate errors into gene-based rare variant tests, explicit modeling of genotype error structures is needed. To do this, precise error models for genotype calling algorithms are needed. Currently, adjustments to, and practical use of, genotype calling algorithms are typically made with a generic sense of reducing errors and improving downstream analysis. Our results provide the basis for making stronger, more direct evaluation of upstream genotype calling algorithms in light of specific power and type I error implications. For example, the results here can be used to determine optimal ratios of *ε*_01_ to *ε*_10_ to minimize power loss—striking a meaningful and justified balance of sensitivity and specificity in the detection of rare alleles. Further work is needed which directly evaluates the decisions made in genotype calling algorithms with regard to their effects on genotype errors and downstream power and type I error implications and the potential development of alternate rare variant tests which explicitly incorporate genotype errors. This work may also include consider of errors involving the rare homozygote which was beyond the scope of our analysis.

Our analysis considers a situation where there is no LD between variants. The general effects of LD on the relationship between genotype errors and test performance are straightforward, while the details are quite complex. In short, the effects of genotype errors will generally be mitigated by LD structure due to (a) the potential for reduced genotype errors when using LD-aware callers and (b) the potential for increased power of multi-marker tests when LD is present between non-causal variants. While this general pattern is true, there is substantial detail related to (a) potential association between genotype error rates and LD structure and (b) potential differences in performance related to the relationship between LD and test statistic choice. Further work is needed to more specifically characterize the impact of LD on the effects of genotype errors.

Consideration of genotype errors in the design of studies is another implication of our work. In particular, we have conclusively demonstrated that power loss will be realized in the presence of non-differential genotype errors. Thus, if a researcher determines that they need *N* subjects to achieve an *a priori* specified level of statistical power, 1 − *β*, in their rare variant analysis, we have demonstrated that, in the presence of non-differential genotype errors, in almost all cases, the actual number of subjects needed is N^*^/*N* > 1. While it is straightforward to see that the value of N^*^/*N* increases in all the same situations that power decreases, tools are needed for researchers to quickly determine how sample size and power estimates should be modified to appropriately account for the impact of genotype errors. The asymptotic power predictions for ***L***_**1**_ and ***J***_**2**_ are provided as a first step toward nearly instantaneous evaluation of the impacts on power and type I error from different types and levels of genotype errors. The main utility in these formulas is in predicting the relative changes in power and type I error from genotype errors. However, even absolute power and type I error predictions were quite accurate in most cases. That said, there is room for improvement if the goal is accurate prediction of absolute power values (e.g., tweaking predictions for a particular variant weighting scheme).

Another important study design consideration relates to differential genotype errors. A growing practice is the use of publicly available databases (e.g., 1000 Genomes Project) as a source of non-diseased subjects since this can substantially reduce study costs. However, in such a case there is no guarantee that the genotype error model is the same in these publicly available databases vs. the error model in the diseased subjects—a situation potentially leading to differential genotype errors and inflated type I errors. The use of the asymptotic equations provided here can give a first level approximation of type I error inflation due to differential genotype errors. As shown, this inflation can be substantial even for modest levels of differential genotype error. Caution should be used when using publically available control samples. While overall methods for controlling the type I error (e.g., genomic control) are available, these methods can substantially reduce power compared to methods with explicitly model, account for or eliminate differential errors. A related issue is that of population stratification which also can inflate the type I error rate. Further work is needed to more fully investigate relationships between population stratification and differential genotype error for rare variant tests of association.

To date only simulation results providing suggestive evidence of the impact of genotyping errors on rare variant tests of association has been available. Our work here, building off of the geometric framework, provides theoretical justification to these patterns. In particular, we demonstrate the potentially substantial impact of common homozygote to heterozygote errors on both power and type I error. The impact of the errors can be intensified depending on the underlying MAF and differential or non-differential nature of the genotype errors, and the test statistic used. Further work is needed to explore additional implications of these results on genotype calling algorithms, study design decisions and rare variant test statistic choice.

### Conflict of interest statement

The authors declare that the research was conducted in the absence of any commercial or financial relationships that could be construed as a potential conflict of interest.
